# Understanding the Post-Treatment Concerns of Cancer Survivors with Five Common Cancers: Exploring the Alberta Results from the Pan-Canadian Transitions Study

**DOI:** 10.3390/curroncol29040218

**Published:** 2022-04-12

**Authors:** Claire Link, Andrea DeIure, Linda Watson

**Affiliations:** 1 Cancer Care Alberta—Alberta Health Services, Calgary, AB T2S 3C3, Canada; claire.link@albertahealthservices.ca (C.L.); andrea.deiure@albertahealthservices.ca (A.D.); 2 Faculty of Nursing, University of Calgary, Calgary, AB T2N 1N4, Canada

**Keywords:** cancer survivors, survivorship care, follow-up care, unmet needs, transitions in care, post-treatment transitions

## Abstract

As the rates of cancer incidence and survival increase in Canada, more patients are living in the post-treatment survivorship phase of their cancer journey. Identifying cancer survivors’ concerns and unmet needs is important so that health care teams can provide relevant information, supports, and resources. Secondary data analysis was carried out on the Alberta patient sample from the 2016 Pan-Canadian Transitions Study survey, designed by the Canadian Partnership Against Cancer. The top concerns for patients treated for five different cancers were examined descriptively and compared. A question about information that patients received post-treatment was also descriptively analyzed. Binary logistic regressions were conducted for each tumour group, using the top three concerns for each group as outcomes and a variety of demographic factors as independent variables. There were 1833 valid respondents in the Alberta sample. Fatigue and anxiety were top concerns for multiple tumour groups. Most patients received more information about treatment side effects than about signs of recurrence and community resources. Within certain tumour groups, younger patients had higher odds of having concerns, particularly anxiety. Awareness of the common and unique concerns experienced by cancer survivors post-treatment enables health care providers to tailor care and resources to help patients manage their symptoms and concerns. These findings address gaps in knowledge around the cancer survivorship phase and may be applicable to cancer programs and primary care providers in Alberta and beyond.

## 1. Introduction

Cancer patients experience unique concerns throughout their cancer journey, and identifying these concerns is critical to providing tailored and comprehensive care. With the survival rate for many cancer types increasing in Canada [1], more patients are completing their treatment, making it essential to understand the challenges experienced by patients in the post-treatment survivorship phase of their cancer journey [2,3]. Many cancer treatments lead to late and long-term physical challenges and symptoms [4,5,6]. Mental health concerns, predominantly anxiety and depression, are also prevalent [7,8]. This points to the fact that this post-treatment patient population often requires specialized resources and services. Research on the concerns and challenges of cancer survivors is increasing, with the United Kingdom’s National Cancer Survivorship Initiative (NCSI) making considerable progress in this area [9,10]. Research in Canada, and within-province research in particular, was scarce prior to 2016 (see Campbell et al. 2011 as an early exception [11]), when the Canadian Partnership Against Cancer (CPAC) undertook a national survey of cancer survivors across the country. 

### 1.1. Experiences of Cancer Patients in Transition Study

A federally funded organization, CPAC considers survivorship a key area for improving experiences, quality of care, and outcomes for cancer patients. In 2016, CPAC administered an innovative national survey called the Experiences of Cancer Patients in Transition Study (commonly referred to as the “Transitions Study”) to cancer survivors across Canada [12]. The goal was to identify key gaps in care and common unmet needs within this population and gain a deeper understanding of the experiences of patients in this phase of their cancer journey. Development of the survey was guided by an extensive literature review, along with consultations with different stakeholders, including cancer survivors [13]. The survey was designed through an iterative process and evaluated with fifteen cancer survivors using cognitive interviewing, followed by performance testing with 96 survivors [13]. 

Surveys were mailed to over 40,000 cancer survivors who were between one- and three-years post-treatment, across all ten Canadian provinces [13]. Ethics approval and survey administration were coordinated by research teams within each province, based on the criteria outlined by CPAC. Participants in all provinces except Quebec had the option of completing the mailed paper survey or an online version, access to which was provided in the mailed information package [13]. In order to survey survivors one to three years beyond their primary cancer treatment, the inclusion criteria were limited to individuals diagnosed with early stage, non-metastatic breast, prostate, colorectal, and melanoma cancers, as well as certain hematological cancers. Additionally, for survivors between 18 and 29 years of age, all non-metastatic cancers were eligible as well as metastatic testicular cancer (for additional details on the survey design, see Fitch et al. 2020 [13]). Since the survey’s completion, many analyses have explored the experiences of transitioned patients in the national sample (see [13,14,15,16,17]—among others). However, to date no studies have focused on provincial-level data to identify the unmet needs of cancer patients at a local level, which could directly inform changes within a provincial cancer program while also providing considerations for other provincial and national cancer organizations.

### 1.2. Cancer Care and Transitions in Alberta

Supporting safe and effective post-treatment transitions for cancer patients and their care teams is a priority in the province of Alberta and is one way to improve patients’ survivorship experiences while creating capacity within the cancer program. The provincial cancer program, Cancer Care Alberta (CCA), has conducted several transitions initiatives since 2013, with the goal of ensuring that patients receive comprehensive follow-up care within and outside of the cancer program, and that patients’ post-treatment concerns can be effectively self-managed or handled by their primary care or cancer care provider [18]. In order to ensure smooth transitions for patients as management of their care shifts between cancer care and primary care, it is crucial to understand what concerns cancer survivors commonly experience and provide appropriate programing, information and supports to help manage these concerns. CCA does not have a systematic way of tracking patients who are transitioned out of the cancer program and as such, prior to the Transitions Study, a large-scale provincial survey focusing on the needs of cancer survivors had not been undertaken.

Although many factors impact the symptoms and concerns that a cancer patient experiences, cancer type is inevitably a heavily influencing factor. Within CCA, care is provided by teams within 13 broad cancer groupings called tumour teams [19]. The organization of care providers into these tumour groupings allows health care professionals to specialize their knowledge and expertise to the needs of specific cancer patient populations. While it is important to understand the common concerns of all cancer survivors, it is equally important to understand what symptoms and concerns are most prevalent in each tumour group so that tumour teams can focus on the needs and issues most relevant to their patients. Specifically, it would be helpful for tumour teams to be aware of the most common challenges encountered by their patients post-treatment, so they can provide appropriate information, supports, and resources to patients prior to their transition from the cancer program. Primary care and community cancer support organizations would also benefit from an awareness of the common and unique concerns experienced by cancer survivors, as they will be required to support a growing proportion of survivors’ post-treatment care needs over the coming years [20]. While other studies have separately examined the unmet needs of cancer survivors by age [13,14,15], no analysis has focused on specific tumour groups or cancer types. Without knowing the unique concerns for each group, it is difficult to implement changes in practice to ensure that the needs of transitioned patients can be met, regardless of the setting in which patients are receiving care.

This study aimed to identify symptoms and concerns prevalent in different populations of cancer patients in Alberta, grouped by common tumour types. A secondary purpose was to identify whether key demographic factors influenced concerns within each tumour group, or if concerns were common to patients of varying demographic backgrounds. The findings of this study will be shared with the provincial cancer program as well as primary care and community organizations throughout Alberta, to increase awareness and help ensure that different care teams are equipped with the knowledge necessary to safely transition patients into post-treatment survivorship and support their well-being over time. The findings could also be used by cancer programs, primary care providers, and community agencies in other jurisdictions, to help address the knowledge gap in the area of cancer survivorship. This is the first study to take a detailed look at any provincial-level data from the Transitions Study.

## 2. Materials and Methods

For this study, secondary data analysis was carried out on the Alberta sample from the CPAC’s Pan-Canadian Transitions Study survey (2016). All data was analyzed using IBM SPSS Statistics software (Version 25.0). The Alberta study was approved by the Health Research Ethics Board of Alberta—Cancer Committee (HREBA-CC). The survey data were collected over a period of 18–19 weeks in Alberta [12]. Approximately 5600 surveys were sent out across the province. Eligible recipients were determined by their inclusion in the Alberta Cancer Registry, which maintains information on all cancer patients diagnosed in Alberta, as well as through examination of administrative data within CCA. Due to limitations with the registry’s processes and within the administrative data, it is difficult to clearly identify patients who have experienced disease progression or completed treatment. As a result, the sampling strategy focused on selecting early-stage cancer patients who were diagnosed one to three years prior to the survey, based on the logic that early-stage cancer treatments are usually curative and in these populations, treatment protocols are usually complete within one year from diagnosis. However, there was no way to exclude patients who were still on treatment due to disease progression or therapeutic need. These limitations in the data will potentially be minimized once the provincial cancer program shifts to a new electronic medical record in late 2022. However, these data limitations impacted the sample selection in Alberta, resulting in some respondents completing the survey who did not meet the eligibility criteria. Similar eligibility issues also impacted data collection in other provinces [13]. As all respondents still had valuable insights on cancer survivorship, and literature in this area is still scarce in Canada, all completed surveys were included in the sample. 

### 2.1. Tumour Group Selection

In order to examine concerns specific to patients with different cancer types, we divided the sample into five tumour groups, based on respondents’ answers to the survey question: *“What type of cancer did you have?”*. There were 17 options provided as well as an “other” option for patients to write in a cancer that was not listed. We sorted responses into the following tumour groups: breast, gastrointestinal, genitourinary, cutaneous, and hematological, as these represented the largest tumour groups in the Alberta sample. The remaining responses were grouped as “other”. Table 1 shows a breakdown of which cancer types were included in each tumour group, along with the associated percentages. As 100% of cutaneous patients were treated for melanoma, this grouping is referred to as “melanoma” from here forward. Respondents who left the question about cancer type blank were included as “other”. The survey was only sent to patients registered in the Alberta Cancer Registry, so it was assumed these respondents simply skipped the question by mistake. Respondents who indicated more than one type of cancer were also included as “other,” as it would have been impossible to determine which cancer influenced their responses more (in terms of the challenges they experienced after transitioning). 

### 2.2. Descriptive Analyses

#### 2.2.1. Demographic Variables

The survey included a series of basic demographic questions asking patients to report characteristics such as their sex, age group, and level of education, as well as questions about self-rated health and details of their cancer (including metastases, treatment, etc.). It should be noted that responses regarding disease-specific information, such as treatment details, were not checked against CCA’s administrative records for accuracy. A series of demographic factors were descriptively analyzed to provide an overview of the Alberta sample. The selection of demographic variables was guided by those included in several publications on the national dataset [13,14,15], to allow for comparability. The demographic variables included were sex, age group, marital status, education, annual household income, employment status, residence (rural or urban), tumour group(s), metastases (yes/no/unsure), time since last treatment, type of treatment received, number of comorbidities (including conditions such as diabetes, arthritis, and hypertension), self-rated physical and emotional health, and self-rated quality of life. 

#### 2.2.2. Changes and Challenges in Survivorship

A series of questions asked respondents about the physical, emotional, and practical changes and challenges they experienced in the post-treatment phase. For each item, respondents were asked, *“How much was this a concern for you?”*. Respondents could select whether their concern was “big”, “moderate”, or “small” or indicate that the item was “not a concern”. For the purposes of this analysis, all three levels of concern were grouped to indicate the total percentage of respondents with each concern. Respondents who did not answer the question were removed from the analysis for that question, leaving only valid responses.

#### 2.2.3. Information after Completing Cancer Treatment

Similar descriptive analyses were conducted on a question that asked respondents about information they had received on various topics related to their post-treatment cancer journey. This question was selected as this information is directly relevant to care teams. The question asked, “*In general, how much do you agree or disagree with the following statements about the information you were given after completing cancer treatment?*”. We focused on information about treatment side effects, signs of recurrence, and community resources. Respondents could select an answer ranging from “strongly disagree” to “strongly agree”, indicating whether they felt they had received sufficient information on each topic. “Strongly agree” and “agree” were grouped to indicate all positive responses. Respondents who did not answer the question or who selected “N/A” were excluded from that question’s analysis. The valid percentage of agreeable respondents was calculated. These analyses were carried out for each tumour group.

### 2.3. Multivariable Binary Logistic Regression Analysis

In order to determine whether the top concerns of patients in each tumour group were influenced by certain demographic factors, binary logistic regressions were run for each tumour group. Three models were run for each group, with the dependent variable for each being the presence of one of the top three concerns for that group (coded as 0 = not concerned, 1 = concerned). Multiple demographic factors were included as independent variables. We wanted to include the same demographic factors in each model, and due to varying sample sizes between the tumour groups, we chose to limit the number of independent variables included, to ensure that even the smallest group had sufficient responses for the analyses. Demographic factors were selected based on the quality of data and the relevance of each factor to clinical practice. The selected demographic factors were age, sex, marital status, rurality, time since last treatment, and number of comorbidities. Sex was excluded from the breast and genitourinary analyses due to these groups being 99.4% female and 99.5% male, respectively. Significance was set a priori at *p* < 0.05.

## 3. Results

### 3.1. Sample Characteristics

In total, of the 5600 surveys administered in Alberta, there were 1834 respondents. This gave a response rate of 32.8%, effectively equal to the national response rate [12]. One respondent indicated that they did not have cancer and was removed from the study, leaving 1833 valid respondents for the analyses. Demographic characteristics for the sample are presented in Table 2. There were slightly more female (51.9%) than male (47.8%) respondents and more than half were aged 65 and older (56.8%). More than one-third had received surgery as their only treatment (34.8%), and nearly three-quarters had been off treatment of any kind for at least one year (72.6%), clearly identifying them as being in the survivorship phase. The most common tumour groups were breast (26.2%), gastrointestinal (21.3%), and genitourinary (20.6%). Most respondents (77.9%) did not report metastases, which was expected as the sampling criteria for the survey was intended to exclude most metastatic cancers. Overall, most respondents rated their physical health (76.2%), emotional health (74.5%), and quality of life (84.0%) as good or very good.

### 3.2. Physical, Emotional, and Practical Concerns by Tumour Group

The survey asked respondents to rate their concern for nine physical symptoms, six emotional symptoms, and five practical issues. Results are presented for each tumour group (results for the “other” grouping are not presented). Looking first at physical concerns (Table 3), we see that “fatigue/tiredness” was a concern for the majority of respondents in each tumour group aside from melanoma (30.5%), ranging from 52.6% of respondents with genitourinary cancer to 91.2% of respondents with hematological cancer. Fatigue was the highest-rated physical concern for breast, melanoma, and hematological respondents. The most common physical concern for gastrointestinal cancer patients was “gastrointestinal problems” (64.6%), and for genitourinary patients the top physical concern was “changes in sexual activity/function” (77.8%). 

Turning to the emotional domain (Table 4), we see that “anxiety/stress/worry about cancer returning” was a concern to the majority of patients in all tumour groups, ranging from 56.0% of genitourinary patients to 76.7% of hematological patients. This was the top emotional concern for patients in all tumour groups except genitourinary. The most comment concern for genitourinary patients was “changes in sexual intimacy” (71.6%).

Finally, looking at the practical domain, we see generally fewer patients in each tumour group indicating concerns (Table 5), compared to the percentage of concerned respondents in the physical and emotional domains. “Returning to work/school” was the top concern in the breast (30.0%), gastrointestinal (22.8%), genitourinary (17.4%), and hematological (37.5%) groupings. The most common concern for melanoma patients was “difficulty getting health/life insurance”, although only 15.4% of patients indicated this as a concern. 

Overall, breast and hematology patients had the same three highest concerns, two physical and one emotional: fatigue, anxiety, and “changes to concentration/memory”. Melanoma patients’ top three concerns were all emotional: anxiety, depression, and “changes in body image”. Gastrointestinal patients’ top concern was “gastrointestinal problems”, followed by fatigue and anxiety. Finally, genitourinary patients had a more unique set of top concerns: “changes in sexual activity/function”, “changes in sexual intimacy”, and “bladder/urinary problems”.

### 3.3. Information after Completing Cancer Treatment

**Figure 1:**
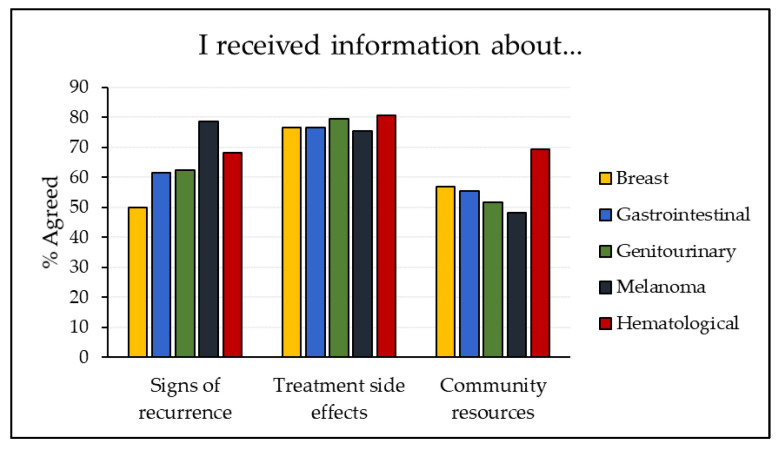
Information after Completing Cancer Treatment (by tumour group).

Figure 1 presents descriptive results of the informational questions for each of the five tumour groups, again excluding “other”. More patients agreed that they had received information about “treatment side effects” than about “signs of recurrence” or “community resources,” with the exception of patients treated for melanoma, who indicated higher agreement regarding information about recurrence. Among patients treated for melanoma, gastrointestinal, and genitourinary cancers, fewer indicated receiving information about resources than about side effects and recurrence. Across all five tumour groups, respondents with melanoma had the highest agreement for “signs of recurrence” and the lowest for “treatment side effects” and “community resources”.

### 3.4. Binary Logistic Regressions by Tumour Group

Three binary logistic regression models were run for each tumour group, one with each of the group’s top three concerns as the outcome. Odds ratios and associated significance values are presented in Table 6, Table 7, Table 8, Table 9 and Table 10. 

In the breast tumour group (Table 6), fatigue, anxiety, and concentration/memory concerns were each included as the outcome in one of the models. Respondents aged 18–44 and 44–64 had significantly higher odds of experiencing all three of these concerns, compared to respondents aged 65 and older, controlling for all included covariates. Patients who responded that they did not receive cancer treatment had significantly lower odds of experiencing both physical symptoms, fatigue, and concentration/memory issues, compared to those who indicated they had received treatment 1–3 years ago. Patients who received treatment more recently (less than one year ago) had significantly lower odds of experiencing concentration/memory concerns that patients who were treated 1–3 years ago. 

Age was also a significant factor in the melanoma grouping (Table 7), with respondents aged 18–44 having significantly higher odds of being concerned with anxiety and depression, compared to respondents aged 65 and older, and 44–64-year-old respondents having significantly higher odds of experiencing anxiety, depression, and changes in body image. Comorbidities also impacted the concerns of melanoma patients, with respondents with two or more comorbidities having significantly higher odds of experiencing anxiety and depression, compared to those with no comorbidities. 

In the regression models for respondents with gastrointestinal cancer (Table 8), males had significantly lower odds of being concerned with gastrointestinal problems than females. Males and females did not significantly differ on the other two outcomes, fatigue and anxiety. Respondents who indicated not receiving treatment had significantly lower odds of experiencing gastrointestinal or fatigue-related concerns, compared to those who reported completing treatment 1–3 years ago. 

Among respondents with genitourinary cancer, those aged 44–64 had significantly higher odds of having concerns about sexual activity than those aged 65 and older (Table 9); 18–44-year-olds had significantly lower odds than those aged 65 and older of having bladder/urinary concerns. We also see some significant results with marital status and rurality in this tumour group. Respondents who were not married or partnered had significantly lower odds of being concerned with sexual activity and sexual intimacy, compared to married respondents. Rural respondents had significantly higher odds of being concerned with sexual intimacy, compared to urban patients.

Finally, turning to the hematology models, we see only two significant findings across all three models, both of which correspond to the model with anxiety as the outcome variable (Table 10). Respondents aged 18–44 had significantly higher odds of being concerned with anxiety, compared to respondents aged 65 and older. Respondents with two or more comorbidities also had significantly higher odds of experiencing anxiety, compared to those with no comorbidities. 

## 4. Discussion

The survivorship phase of a patient’s cancer journey is a time of change and adaptation as patients complete active cancer treatments, transition back to their primary care providers for follow-up care, and leave their sick role to return to their everyday lives and responsibilities. The end of treatment does not mean the end of symptoms, side effects, and concerns, and identifying patient needs in this phase is essential to ensure that patients are provided with the knowledge, support, and resources to manage these needs in the post-treatment phase of their cancer journey. The findings of this study support this, demonstrating that a majority of respondents experienced physical and emotional concerns in the survivorship phase, with a smaller percentage experiencing practical issues. Greater attention should be given to the identification and management of late effects of cancer and associated treatments, to ensure that survivors and their care teams are prepared if and when these issues arise. 

### 4.1. Common Concerns

Examining the top concerns for patients in each tumour group, we see that patients do not all have the same challenges, although there are some common concerns across most tumour groups such as fatigue and anxiety. This points to the importance of preparing cancer patients moving into the post-treatment phase with information on managing both physical and emotional symptoms, as both are likely to be prevalent. Ambulatory cancer programs as well as primary care and community organizations may want to dedicate resources or funding to enhance services related to managing these symptoms, as they are common across cancer types. Anxiety was identified as a top concern for patients in all tumour groups except genitourinary cancer. Existing research suggests that anxiety is high among cancer survivors, often largely due to the ever-present fear of recurrence [8,21,22]. Age significantly impacted the odds of having concerns with anxiety for patients in the breast, melanoma, and hematological tumour groups. Younger patients (aged 18–44 and 44–64) had significantly higher odds than patients aged 65 and older. In particular, the youngest age group’s odds were dramatically higher, with an odds ratio around seven for hematology and melanoma patients and an odds ratio greater than eight for breast cancer patients. This fits with literature demonstrating that younger patients, particularly those in the 15–39 age range (commonly called Adolescents and Young Adults, or AYAs), regularly experience heightened anxiety and fear of recurrence [23,24]. The distinct needs and experiences of AYA cancer survivors are increasingly being recognized, and AYA-specific resources are being developed by cancer programs within and beyond Canada [25,26,27]. Tumour teams and primary care providers may benefit from gaining awareness of these resources and ensuring that their patients are aware as well. Notably, while anxiety was also a top concern for gastrointestinal patients in this study, there were no significant differences based on the included demographic factors. 

Fatigue was a top concern for patients in three of the five examined tumour groups. Like anxiety, fatigue has long been identified as a common issue among cancer survivors, as many treatments lead to long-term fatigue and tiredness [28,29,30]. This symptom is challenging because it requires considerable self-management on the part of patients, especially post-treatment, as patients move out of their sick role and work to reclaim their everyday lives. Patients may use a variety of strategies including physical activity, mindfulness exercises (such as yoga), and different kinds of therapeutic interventions [31]. Cancer care teams should ensure their transitioning patients have a variety of information related to fatigue self-management and community resources so that patients are aware of different strategies and programs available and can determine what works best for them. Additionally, patients may benefit from health coaching from their care providers to ensure they feel confident and knowledgeable about their self-management responsibilities in the post-treatment phase. Studies have shown that dedicated health coaching has measurable quality of life and symptom-specific improvements for cancer survivors [32]. A study in China demonstrated significant reductions in cancer-related fatigue specifically for pediatric cancer patients who received home-visit health coaching [33]. While home visits may not be possible for adult cancer survivors, given the much larger population size, health coaching in other forms should be considered by cancer care, primary care, and community programs, particularly for fatigue and other symptoms for which self-management is critical. In the current study, among breast cancer survivors, younger survivors had significantly higher odds of being concerned with fatigue than older patients; however, age did not significantly influence fatigue concerns for gastrointestinal or hematological patients. Younger breast patients may be more concerned about fatigue due to lifestyle differences tied to their younger age, or they may have less awareness of relevant resources available to them. Health care providers with younger patients, and younger breast patients in particular, may want to take care to check in with these patients about their fatigue and associated self-management techniques, to ensure their concerns are being adequately addressed. 

### 4.2. Tumour Group-Specific Concerns

While fatigue and anxiety were common concerns across most tumour groups, there were multiple unique issues as well. Unsurprisingly, patients treated for gastrointestinal cancer were more concerned with gastrointestinal problems than were other patients. Interestingly, within this tumour group, male patients had about half the odds of having these concerns compared to female patients. This could serve as a flag to gastrointestinal care teams that their female patients may benefit from additional resources to help manage their concerns. Genitourinary cancer patients differed considerably from the other patient groups, with their three highest concerns—sexual function, sexual intimacy, and bladder/urinary problems—all being unique to this group. Tumour teams and primary care providers caring for genitourinary patients may want to use a tailored approach when supporting these patients post-treatment, as general survivorship resources may not be sufficient to address their distinct concerns. Importantly, previous studies have identified that sexual health and sexual concerns are often an area of unmet need for cancer survivors [34,35,36], and the findings of the current study support this. This suggests that patients, and perhaps particularly genitourinary patients, would benefit from additional information on these topics prior to their transition. In contrast to the unique top concerns of this group, patients in the breast and hematological tumour groupings had the same top three concerns, in the same order. This knowledge could prompt cancer programs and community organizations to develop more general resources that can be used by patients of any cancer diagnosis.

Interestingly, while the multivariable analyses revealed key demographic factors influencing concerns within certain turmour groups, they also revealed that demographic factors were not as relevant within other groups. Hematology is the best example of this, with only two significant findings across all three models. This suggests that the issues most concerning to these patients are quite common across the majority of patients with hematological cancers—in other words, their concerns are more related to the cancer itself, rather than demographic factors. This is supported by the descriptive results. Looking at the three top concerns for hematological patients, we see that a higher percentage of patients in this grouping endorsed fatigue (91.2%), anxiety (76.7%), and concentration/memory (63.7%) than in any other tumour group. The percentage of hematology respondents who indicated concern with fatigue was the highest percentage of respondents indicating any concern, across all tumour groups, for any of the 20 concerns included in the survey. Providers who care for hematological patients should ensure to discuss fatigue, anxiety, and concentration/memory with each patient, as it is likely that these issues will be of concern regardless of a patient’s demographic background.

### 4.3. Information after Completing Cancer Treatment

The information questions have direct practice implications, highlighting areas where clinicians in different tumour teams could work to provide additional information to prepare patients for their transition back to their community for follow-up care. In the Alberta sample, a higher percentage of patients agreed that they had received information about treatment side effects than about other topics (with melanoma patients as an exception). This general pattern is reflected in the national adult survey sample, with a previous study finding that 81% of respondents agreed that they received information about treatment side effects, while only 62% and 58% agreed that they received information about signs of recurrence and community resources, respectively [13]. 

Cancer care providers likely, and understandably, have greater awareness of treatment side effects and signs of recurrence than of community-based resources available to patients. It is important for cancer programs to promote awareness of relevant community resources to different tumour teams so that they can share this information with their patients. A higher percentage of patients treated for hematological cancers agreed that they had received information about community resources; with this in mind, the hematology tumour team in Alberta could potentially be looked at as a positive example for other tumour teams. In addition to resource information, it is critical that tumour teams provide more information about signs of recurrence to both patients and primary care teams, as there is considerable research demonstrating that the fear of recurrence is a major source of anxiety for patients in the survivorship phase [21,37,38]. It would be beneficial for cancer programs to develop tailored patient education materials for each tumour group, including general and disease-specific resources and information, which could be distributed to patients before their transition from the cancer program. It would also be helpful for similar information to be shared with primary care teams, as there is strong evidence suggesting that there are often gaps in knowledge in this area for primary care providers [20].

It should be noted that, across patients treated for the five included cancer types, practical concerns such as “returning to work/school” or “paying health care bills,” were not top concerns. However, patients still encounter practical challenges, as highlighted in Table 5. Patients could benefit from being provided with information and resources to manage their practical issues in addition to their symptoms [39]. Much of the practical information could likely be general rather than tumour-specific.

### 4.4. Study Limitations

This study is necessarily limited by the data collected from the Transitions Study. Although members of the study team were involved in the administration of the Alberta portion of the survey, we did not have a role in designing the survey itself. The physical, emotional, and practical challenges included in the survey may not encompass all concerns that cancer survivors have. There was one open-ended question for each category of concern where respondents could write in additional comments; these were not analyzed in the current study, but analysis of the open-ended responses may be considered in the future. Tumour group selection was made based on the groupings used within CCA and on the number of respondents indicating each cancer type. Some cancer types, such as gynecological cancers, did not have enough respondents to constitute their own grouping, and these respondents were included in the “other” category. However, patients in this mixed group likely have diverse concerns that should also be identified in order to improve their transitions and survivorship care. Analyzing “other” respondents within the larger national sample could be a useful strategy to address this limitation in the future. The sample of patients in this study may not be representative of all Alberta cancer survivors. The majority of respondents had not received treatment for more than one year, and as such their concerns may differ from patients who completed treatment more recently. As mentioned, due to data limitations, the Alberta sample did not exactly match the eligibility criteria for the survey. However, the demographic distribution of the Alberta sample closely resembles that of the larger national sample [13], suggesting that this was a limitation of the survey as a whole rather than an Alberta-specific issue. The decision to focus on the Alberta sample in this study, rather than the larger national sample, limits the generalizability of the findings. However, this was an intentional choice, as the goal of this study was to directly inform practice within Cancer Care Alberta, and therefore the provincial-level data was of most relevance. Importantly, although there are multiple publications on the national data, none followed a similar approach to that of the current study and examined concerns by tumour group or cancer type. We will consider conducting a similar tumour group analysis using the larger national sample in the future. Finally, it is important to note that this survey was conducted in 2016, several years before the COVID-19 pandemic and the associated worldwide impacts. The concerns of cancer survivors may have changed somewhat since the start of the pandemic; anxiety may be heightened, or patients may be more concerned with relationships with friends or family, resulting from the social distancing policies put in place across the world. These are only suggestions, but it should be acknowledged that the results of this study would likely differ somewhat if the survey was repeated today.

## 5. Conclusions

Cancer patients experience a multitude of challenges during the survivorship phase of their cancer journey. While it can be difficult for care teams to provide comprehensive information on every possible symptom a patient may experience, understanding common concerns is key to ensuring smooth transitions from cancer care to primary care once treatment is complete, as well as high-quality survivorship care in the community setting. The type of cancer that a patient was treated for has considerable influence on the symptoms and concerns that they will experience post-treatment, and identifying top concerns in each tumour group enables care teams to provide tailored support, information, and resources to best meet patients’ needs. In addition, acknowledging that some concerns are common across all tumour groups could aid cancer care, primary care, and community programs in developing and providing more general education, resources, and supports. The findings from this study should serve as a flag to health care providers of the importance of monitoring the concerns of cancer survivors, as they may experience multiple concerns years after treatment is complete. These findings will be shared within CCA to inform ongoing work to improve transitions in cancer care, and with community and primary care programs. Other ambulatory oncology programs, provincial health care agencies, and community agencies may draw on these findings to gain awareness of unique and common issues in the survivorship phase and, in turn, use this information to increase resource awareness and further program development to help clinicians, and patients, better manage post-treatment concerns.

## Figures and Tables

**Table 1:** Tumour Group Classification.

Breast	Cutaneous ^a^	Gastrointestinal	Genitourinary	Hematological	Other
Breast (100%)	Melanoma (100%)	Colorectal (97.2%)	Prostate (93.1%)	Other blood cancer (33.5%)	Blank (53.6%)
		Stomach/ Esophagus (1.5%)	Testicular (4.0%)	Hodgkin’s lymphoma (25.9%)	Other Cancer ^b^ (25.9%)
		Abdominal/ Appendix (1.0%)	Bladder (2.1%)	Diffuse B-cell lymphoma (25.9%)	2 or more cancers (20.5%)
		Liver/Hepatic (0.3%)	Renal/ Kidney (0.8%)	Acute myelogenous or lymphocytic leukemia (14.7%)	

a: As “cutaneous” included melanoma patients only, this group is referred to as “melanoma” from here forward. b: “Other cancer” includes: Brain/central nervous system, gynecological, sarcoma, thyroid, bone, lungs/respiratory, oral/tongue, throat, and ganglion cancers.

**Table 2:** Alberta sample demographics.

Characteristic	*N*	%
Sex		
Male	876	47.8
Female	951	51.9
Blank	6	0.3
Age		
18–44	144	7.9
45–64	643	35.1
65+	1042	56.8
Blank	4	0.2
Marital Status		
Not married/partnered (single, divorced, separated, or widowed)	463	25.3
Married/partnered	1353	73.8
Blank	17	0.9
Education		
High school or less	551	30.1
College or university (below Bachelor’s level)	729	39.8
Bachelor’s degree or higher	498	27.2
Blank	55	3.0
Annual Household Income		
Less than $25,000	177	9.7
$25,000 to >$50,000	349	19.0
$50,000 to >$75,000	288	15.7
$75,000 or more	598	32.6
Blank	421	23.0
Employment Status		
Employed	674	36.8
Not in paid employment (homemaker, student, retired)	1041	56.8
Unemployed	79	4.3
Blank	39	2.1
Residence		
Rural	521	28.4
Urban	1269	69.2
Blank	43	2.3
Tumour Group ^a^		
Breast	481	26.2
Melanoma	249	13.6
Gastrointestinal	390	21.3
Genitourinary	379	20.7
Hematological	116	6.3
Other	218	11.9
Metastases		
Yes	161	8.8
No	1428	77.9
Unsure	161	8.8
Blank	83	4.5
Time since treatment		
Less than 1 year	224	12.2
1 to 3 years	890	48.6
More than 3 years	440	24.0
Did not receive treatment	221	12.1
Blank	58	3.2
Type of treatment		
Surgery only	637	34.8
Drug only (chemotherapy or other)	110	6.0
Radiation therapy only	131	7.1
Combination/other	824	45.0
None	66	3.6
Blank	65	3.5
Number of comorbidities		
None	682	37.2
1	617	33.7
2	467	25.5
Blank	67	3.7
General physical health		
Very good/good	1397	76.2
Fair	358	19.5
Very poor/poor	69	3.8
Blank	9	0.5
General emotional health		
Very good/good	1366	74.5
Fair	322	17.6
Very poor/poor	65	3.5
Blank	80	4.4
Overall quality of life		
Very good/good	1539	84.0
Fair	258	14.1
Very poor/poor	32	1.7
Blank	4	0.2

a: Tumour group does not have “Blank” respondents, as these were included as “other” (see Table 1 for details).

**Table 3:** Respondents’ concerns in the physical domain (by tumour group).

Physical	Breast	Melanoma	Gastrointestinal	Genitourinary	Hematological
	*% (n)* *Concerned*	*% (n)* *Concerned*	*% (n)* *Concerned*	*% (n)* *Concerned*	*% (n)* *Concerned*
Lymphedema	41.0 (191) Total = 466	18.1 (43) Total = 237	12.9 (46) Total = 357	8.1 (28) Total = 347	21.6 (24) Total = 111
Fatigue/tiredness	79.1 (370) Total = 468	30.5 (73) Total = 239	61.8 (230) Total = 372	52.6 (185) Total = 352	91.2 (103) Total = 113
Hormonal/menopause/ fertility	40.0 (186) Total = 465	6.3 (15) Total = 238	8.1 (29) Total = 358	19.1 (65) Total = 340	16.2 (18) Total = 111
Chronic/long-term pain	44.1 (206) Total = 467	17.8 (42) Total = 236	32.7 (117) Total = 358	20.7 (72) Total = 347	33.0 (37) Total = 112
Bladder/urinary problems	20.1 (93) Total = 462	7.6 (18) Total = 238	28.7 (104) Total = 363	70.7 (248) Total = 351	27.9 (31) Total = 111
Gastrointestinal problems	25.6 (119) Total = 464	9.7 (23) Total = 236	64.6 (239) Total = 370	29.0 (100) Total = 345	48.6 (54) Total = 111
Nerve problems	47.4 (221) Total = 466	29.0 (69) Total = 238	33.6 (123) Total = 366	19.0 (66) Total = 347	57.7 (64) Total = 111
Changes to concentration/memory	56.0 (262) Total = 468	17.4 (41) Total = 236	33.6 (123) Total = 366	22.7 (77) Total = 339	63.7 (72) Total = 113
Changes in sexual activity/function	35.3 (165) Total = 468	11.0 (26) Total = 236	27.9 (102) Total = 365	77.8 (277) Total = 356	34.8 (39) Total = 112

**Table 4:** Respondents’ concerns in the emotional domain (by tumour group).

Emotional	Breast	Melanoma	Gastrointestinal	Genitourinary	Hematological
	*% (n)* *Concerned*	*% (n)* *Concerned*	*% (n)* *Concerned*	*% (n)* *Concerned*	*% (n)* *Concerned*
Depression/ sadness	52.1 (228) Total = 438	31.8 (68) Total = 214	40.2 (137) Total = 341	44.0 (149) Total = 339	53.4 (55) Total = 103
Anxiety, stress, or worry about cancer returning	76.3 (334) Total = 438	68.1 (145) Total = 213	61.5 (211) Total = 343	56.0 (191) Total = 341	76.7 (79) Total = 103
Changes in relationships (family)	31.5 (149) Total = 473	17.9 (43) Total = 240	25.8 (95) Total = 368	41.1 (147) Total = 358	38.6 (44) Total = 114
Changes in relationships (friends)	24.5 (115) Total = 469	12.5 (30) Total = 240	21.4 (79) Total = 369	14.9 (53) Total = 356	31.6 (36) Total = 114
Changes in body image	55.1 (259) Total = 470	31.7 (76) Total = 240	31.7 (117) Total = 369	25.1 (88) Total = 351	43.0 (49) Total = 114
Changes in sexual intimacy	37.6 (175) Total = 466	13.4 (32) Total = 238	26.0 (95) Total = 365	71.6 (257) Total = 359	34.2 (39) Total = 114

**Table 5:** Respondents’ concerns in the practical domain (by tumour group).

Practical	Breast	Melanoma	Gastrointestinal	Genitourinary	Hematological
	*% (n)* *Concerned*	*% (n)* *Concerned*	*% (n)* *Concerned*	*% (n)* *Concerned*	*% (n)* *Concerned*
Returning to work/school	30.0 (140) Total = 466	10.7 (26) Total = 243	22.8 (84) Total = 368	17.4 (62) Total = 356	37.5 (42) Total = 112
Getting to/from appointments	21.4 (100) Total = 467	12.8 (31) Total = 243	19.8 (73) Total = 368	12.9 (46) Total = 356	26.8 (30) Total = 112
Taking care of family members	17.7 (83) Total = 468	8.2 (20) Total = 243	12.2 (44) Total = 362	8.7 (31) Total = 356	21.8 (24) Total = 110
Difficulty getting health/life insurance	19.7 (92) Total = 466	15.4 (37) Total = 240	15.6 (57) Total = 365	14.7 (52) Total = 353	19.8 (22) Total = 111
Difficulty paying health care bills	18.5 (87) Total = 471	13.2 (32) Total = 243	16.6 (61) Total = 368	16.3 (58) Total = 355	30.0 (33) Total = 110

**Table 6:** Odds ratios from binary logistic regressions for breast patients.

	Fatigue		Anxiety		Concentration/Memory	
	Odds Ratio	Sig.	Odds Ratio	Sig.	Odds Ratio	Sig.
*Age*						
18–44	**6.156**	**0.019**	**8.212**	**0.006**	**6.781**	**0.000**
44–64	**2.083**	**0.007**	**2.922**	**0.000**	**2.437**	**0.000**
65+ *	-	-	-	-	-	-
*Marital Status*						
Not married/partnered	0.638	0.106	0.588	0.054	0.950	0.830
Married/partnered *	-	-	-	-	-	-
*Rurality*						
Rural	1.062	0.829	1.248	0.426	0.848	0.468
Urban *	-	-	-	-	-	-
*Education*						
High school or less	**0.516**	**0.022**	0.660	0.153	1.029	0.910
College/some university *	-	-	-	-	-	-
Bachelor’s degree or higher	0.985	0.963	1.267	0.457	1.152	0.574
*Time since treatment*						
Less than 1 year	0.831	0.672	2.618	0.067	**0.481**	**0.036**
1 to 3 years *	-	-	-	-	-	-
3 years or more	0.690	0.186	1.276	0.390	1.017	0.941
Did not receive treatment	**0.272**	**0.011**	0.476	0.188	**0.183**	**0.010**
*Comorbidities*						
None *	-	-	-	-	-	-
1	1.807	0.053	1.486	0.190	1.275	0.328
2 or more	**2.083**	**0.025**	1.673	0.109	1.595	0.080
*Model N*	437 (44 missing)		409 (72 missing)		438 (43 missing)	

Note: Sex was excluded from this analysis, as 99.4% of the breast sample was female. * Reference group. Bold type indicates significant findings (*p* < 0.05).

**Table 7:** Odds ratios from binary logistic regressions for melanoma patients.

	Anxiety		Depression		Changes in Body Image	
	Odds Ratio	Sig.	Odds Ratio	Sig.	Odds Ratio	Sig.
*Age*						
18–44	**7.009**	**0.020**	**8.670**	**0.001**	2.119	0.195
44–64	**2.877**	**0.004**	**2.036**	**0.050**	**2.577**	**0.004**
65+ *	-	-	-	-	-	-
*Sex*						
Male	0.584	0.108	0.697	0.285	0.618	0.120
Female *	-	-	-	-	-	-
*Marital Status*						
Not married/partnered	1.964	0.120	1.375	0.404	1.115	0.760
Married/partnered *	-	-	-	-	-	-
*Rurality*						
Rural	1.424	0.355	0.992	0.983	1.387	0.318
Urban *	-	-	-	-	-	-
*Education*						
High school or less	1.205	0.661	1.863	0.132	1.329	0.461
College/some university *	-	-	-	-	-	-
Bachelor’s degree or higher	1.297	0.503	0.995	0.990	1.159	0.686
*Time since treatment*						
Less than 1 year	0.782	0.595	2.333	0.070	1.749	0.194
1 to 3 years *	-	-	-	-	-	-
3 years or more	1.325	0.549	1.120	0.804	1.037	0.931
Did not receive treatment	1.341	0.510	1.074	0.879	0.566	0.204
*Comorbidities*						
None *	-	-	-	-	-	-
1	1.275	0.545	1.958	0.136	1.597	0.228
2 or more	**2.575**	**0.039**	**2.776**	**0.027**	1.840	0.135
*Model N*	204 (45 missing)		205 (44 missing)		228 (21 missing)	

* Reference group. Bold type indicates significant findings (*p* < 0.05).

**Table 8:** Odds ratios from binary logistic regressions for gastrointestinal patients.

	Gastrointestinal Problems		Fatigue		Anxiety	
	Odds Ratio	Sig.	Odds Ratio	Sig.	Odds Ratio	Sig.
*Age*						
18–44	1.104	0.894	0.444	0.280	7.064	0.075
44–64	1.316	0.306	1.000	0.999	1.532	0.121
65+ *	-	-	-	-	-	-
*Sex*						
Male	**0.512**	**0.008**	0.708	0.175	0.764	0.290
Female *	-	-	-	-	-	-
*Marital Status*						
Not married/partnered	0.791	0.387	0.738	0.276	0.717	0.230
Married/partnered *	-	-	-	-	-	-
*Rurality*						
Rural	1.014	0.957	1.351	0.276	1.082	0.775
Urban *	-	-	-	-	-	-
*Education*						
High school or less	1.037	0.895	0.890	0.689	0.673	0.166
College/some university *	-	-	-	-	-	-
Bachelor’s degree or higher	1.140	0.671	0.774	0.411	0.647	0.171
*Time since treatment*						
Less than 1 year	1.188	0.723	0.613	0.290	1.106	0.836
1 to 3 years *	-	-	-	-	-	-
3 years or more	0.745	0.294	**0.485**	**0.012**	0.795	0.427
Did not receive treatment	**0.491**	**0.032**	**0.154**	**0.000**	0.539	0.078
*Comorbidities*						
None *	-	-	-	-	-	-
1	1.156	0.608	1.628	0.092	1.533	0.148
2 or more	0.978	0.940	1.160	0.629	0.790	0.448
*Model N*	338 (52 missing)		337 (53 missing)		313 (77 missing)	

* Reference group. Bold type indicates significant findings (*p* < 0.05).

**Table 9:** Odds ratios from binary logistic regressions for genitourinary patients.

	Sexual Activity/ Function		Sexual Intimacy		Bladder/ Urinary Problems	
	Odds Ratio	Sig.	Odds Ratio	Sig.	Odds Ratio	Sig.
*Age*						
18–44	0.591	0.419	0.529	0.318	**0.035**	**0.002**
44–64	**4.177**	**0.001**	1.435	0.229	1.071	0.810
65+ *	-	-	-	-	-	-
*Marital Status*						
Not married/partnered	**0.381**	**0.007**	**0.312**	**0.000**	0.636	0.192
Married/partnered *	-	-	-	-	-	-
*Rurality*						
Rural	1.778	0.085	**2.013**	**0.019**	1.448	0.205
Urban *	-	-	-	-	-	-
*Education*						
High school or less	0.562	0.084	0.724	0.291	0.767	0.397
College/some university *	-	-	-	-	-	-
Bachelor’s degree or higher	1.169	0.673	1.031	0.922	0.918	0.783
*Time since treatment*						
Less than 1 year	0.485	0.054	**0.466**	**0.027**	0.770	0.466
1 to 3 years *	-	-	-	-	-	-
3 years or more	0.912	0.801	0.764	0.398	1.243	0.514
Did not receive treatment	0.899	0.819	0.900	0.804	0.485	0.065
*Comorbidities*						
None *	-	-	-	-	-	-
1	0.916	0.798	0.721	0.281	0.754	0.344
2 or more	1.132	0.746	0.937	0.848	1.057	0.875
*Model N*	337 (42 missing)		341 (38 missing)		332 (47 missing)	

Note: Sex was excluded from this analysis, as 99.5% of the genitourinary sample was male. * Reference group. Bold type indicates significant findings (*p* < 0.05).

**Table 10:** Odds ratios from binary logistic regressions for hematological patients.

	Fatigue		Anxiety		Concentration/ Memory	
	Odds Ratio	Sig.	Odds Ratio	Sig.	Odds Ratio	Sig.
*Age*						
18–44	1.811	0.630	**6.991**	**0.039**	2.147	0.255
44–64	1.005	0.995	2.677	0.103	1.426	0.462
65+ *	-	-	-	-	-	-
*Sex*						
Male	0.873	0.858	0.603	0.367	0.800	0.614
Female *	-	-	-	-	-	-
*Marital Status*						
Not married/partnered	2.787	0.385	0.564	0.375	1.105	0.854
Married/partnered *	-	-	-	-	-	-
*Rurality*						
Rural	1.143	0.886	1.047	0.944	0.975	0.961
Urban *	-	-	-	-	-	-
*Education*						
High school or less	0.471	0.431	0.539	0.344	0.777	0.625
College/some university *	-	-	-	-	-	-
Bachelor’s degree or higher	0.655	0.687	1.248	0.769	1.642	0.407
*Time since treatment*						
Less than 1 year	2.31 × 10^8^	0.999	6.62 × 10^8^	0.999	4.319	0.085
1 to 3 years *	-	-	-	-	-	-
3 years or more	3.599	0.266	1.179	0.785	2.292	0.134
Did not receive treatment ^a^	-	-	-	-	-	-
*Comorbidities*						
None *	-	-	-	-	-	-
1	1.012	0.988	2.191	0.221	1.079	0.882
2 or more	1.84 × 10^8^	0.998	**6.932**	**0.036**	2.828	0.109
*Model N*	106 (10 missing)		96 (20 missing)		106 (10 missing)	

a: “Did not receive treatment” was not included in the hematology models due to insufficient observations (*n* = 0). * Reference group. Bold type indicates significant findings (*p* < 0.05).

## Data Availability

The aggregated national Transitions Study data is available from the Canadian Partnership Against Cancer, for research, practice, and policy specialists. The data access form can be found here: https://www.partnershipagainstcancer.ca/transition-study/ (accessed on 25 February 2022). Provincial-level data may be available from the Canadian Partnership Against Cancer upon request.
